# Acupuncture combined with SSRIs in the treatment of perimenopausal depressive disorder: A protocol for systematic review and meta-analysis

**DOI:** 10.1097/MD.0000000000028558

**Published:** 2022-01-21

**Authors:** Zhan Gao, Jieqiong Wang, Mingzhou Gao, Tingting Song, Xiangju Gao, Mingqi Qiao, Jie Gao

**Affiliations:** Shandong University of Traditional Chinese Medicine, China.

**Keywords:** acupuncture, depressive disorder, meta-analysis, perimenopausal depressive disorder, perimenopausal period, protocol, selective serotonin reuptake inhibitors, systematic review

## Abstract

**Background:**

Perimenopausal depressive disorder (PDD) is an affective disorder involving endocrine, neurological, immune, which seriously endangers the physical and mental health of human. Selective serotonin reuptake inhibitors (SSRIs) are the current first-line clinical treatment, have limited efficacy and serious side effects. Acupuncture combined with SSRIs therapy has been widely used clinically because it increases efficacy and reduces side effects. There is a lack of high-quality evidence to assess its efficacy and safety. This study evaluated the effectiveness and safety of acupuncture combined with SSRIs in the treatment of PDD by meta-analysis.

**Methods:**

All randomized controlled trials articles about acupuncture combined with SSRIs treatment of PDD will be searched in databases, such as PubMed, Cochrane Library, Web of Science, China National Knowledge Infrastructure, Wan Fang, Wei Pu from the construction of the library to December 16, 2021. According to Cochrane 5.1 Handbook criteria, two researchers independently screened the literature, extracted data, and evaluated the quality of included studies. Meta-analysis was performed by using RevMan 5.4 and STATA 16.0 software.

**Results:**

This study will summarize the current evidence to evaluate the effectiveness and safety of acupuncture combined with SSRIs for the treatment of PDD.

**Conclusion:**

The results of this study will provide clinicians with new treatment ideas and bring benefits to most patients.

**Registration number:**

INPLASY2021120080 (DOI number: 10.37766/inplasy2021.12.0080).

## 1 Introduction

Perimenopause is the physiological transition period from the onset of menstrual disorders to 1 year after menopause in women,^[1–3]^ most women transition to perimenopause at approximately 40 to 60 years of age. Perimenopausal depressive disorder (PDD) is a mood disorder in which women experience persistent depressed mood, anxiety and depressive disorder, and vegetative nervous disorders during perimenopause.^[4,5]^ Women are about twice as likely as men to be depressed, and perimenopausal women are about twice as likely as premenopausal women to be depressed.^[6]^ Studies showed a 36% prevalence of depressive disorder in perimenopausal women,^[7]^ and the trend is increasing year by year. If patients with PDD are not treated promptly or given inappropriate treatment, it increases the risk of suffering and suicide. Selective serotonin reuptake inhibitors (SSRIs) are the first-line treatment for depressive disorder,^[8]^ it is also used clinically for the treatment of PDD.^[9]^ However, patient adherence to SSRIs is low for two main reasons:

1) SSRIs act solely on the serotonin system and do not address the multisystem disorders of PDD;
2) SSRIs have side effects such as gastrointestinal distress, weight changes, sleep disturbances, and sexual dysfunction.^[10–12]^

The combination of acupuncture and SSRIs in the treatment of depressive disorder has been proven to have increased efficacy and reduced side effects, which may provide a new direction for clinical treatment.^[13]^ Acupuncture treatment upholds a holistic concept, so it can regulate and treat PDD at multiple levels, links, and targets, such as regulation of sex hormones, neurotransmitters, hypothalamic-pituitary-adrenal axis, hypothalamic-pituitary-ovarian axis, oxidative stress, signaling pathways, and other cellular events.^[14]^ Acupuncture can compensate for the inability of SSRIs to address multisystem disorders in PDD. Unluckily, acupuncture is difficult to achieve short-term results for severe depressive disorder. Acupuncture as a physical stimulus to accelerate the onset of action of SSRIs and reduce the dose and side effects of antidepressant applications.^[15]^ The combination of acupuncture and SSRIs in the treatment of PDD can take advantage of the wide range of acupuncture effects, and take advantage of the precise pharmacology and specific effects of SSRIs to achieve the effect of increasing efficacy and reducing side effects.^[15]^ Studies showed acupuncture combined with antidepressants had excellent efficacy in treating PDD.^[16]^ There is no high-quality evidence to assess the efficacy and safety of acupuncture in combination with SSRIs. This study investigated the effectiveness and safety of acupuncture combined with SSRIs by meta-analysis to provide clinicians with a better basis for clinical decisions.

## 2 Methods

### 2.1 Study registry

The systematic review protocol was registered on the International Platform of Registered Systematic Review and Meta-analysis Protocols. The registration number: INPLASY2020110072. The consent of this protocol report is based on the Preferred Reporting Items for Systematic Reviews and Meta-Analyses Protocols (PRISMAP) statement guidelines.^[17]^

### 2.2 Literature sources and search

From the construction of the library to December 16, 2021, all randomized controlled trials (RCTs) articles about acupuncture combined with SSRIs treatment of PDD will be searched in databases, such as PubMed, Cochrane Library, Web of Science, China National Knowledge Infrastructure, Wan Fang, and Wei Pub by advanced search. The key search terms used are (Perimenopause or Climacteric or Menopause) And (Depressive Disorder or Mood Disorders) And (SSRIs or Fluoxetine or Paroxetine or Sertraline or Fluvoxamine or Citalopram or Escitalopram) And (Acupuncture or manual acupuncture or electro-acupuncture or scalp acupuncture or auricular acupuncture). The search strategy for PubMed is shown in Table 1.

**Table 1:** Search strategy used in PubMed database.

No.	Search items
#1	Perimenopause [Title/Abstract]
#2	Climacteric [Title/Abstract]
#3	Menopause [Title/Abstract]
#4	#1 or #2 or #3
#5	Depressive disorder [Title/Abstract]
#6	Mood disorders [Title/Abstract]
#7	#5 or #6
#8	Selective serotonin reuptake inhibitors [Title/Abstract]
#9	Fluoxetine [Title/Abstract]
#10	Paroxetine [Title/Abstract]
#11	Sertraline [Title/Abstract]
#12	Fluvoxamine [Title/Abstract]
#13	Citalopram [Title/Abstract]
#14	Escitalopram [Title/Abstract]
#15	#8 or #9 or #10 or #11 or #12 or #13 or #14
#16	Acupuncture [Title/Abstract]
#17	Manual acupuncture [Title/Abstract]
#18	Electro-acupuncture [Title/Abstract]
#19	Scalp acupuncture [Title/Abstract]
#20	Auricular acupuncture [Title/Abstract]
#21	#16 or #17 or #18 or #19 or #20
#22	#4 or #7 or #15 or #21

### 2.3 Eligibility criteria for including studies

The participant, intervention, comparison, and study design (PICOS) principle has been applied in the study design.

#### 2.3.1 Types of studies

We will include clinical RCTs, which will be categorized by language in Chinese and English. Any other types of studies, such as animal studies, case reports, case series and reviews, will be excluded.

#### 2.3.2 Types of patients

Women were diagnosed with PDD. The diagnostic criteria for depressive disorder were in accordance with the Chinese Classification and Diagnostic Criteria of Mental Disorders, 3rd edition (CCMD-3), American Psychiatric Association DSM-5, American Psychiatric Association's Diagnostic and Statistical Manual of Mental Disorders; diagnostic criteria for perimenopause in accordance with the Diagnostic Criteria for Gynecological Diseases, the diagnostic criteria for TCM evidence should be in accordance with the “Internal Medicine of Chinese Medicine” or “Diagnostic and Efficacy Criteria for TCM Medical Evidence”.

#### 2.3.3 Types of interventions

##### 2.3.3.1 Experimental group

All patients used received acupuncture combined with SSRIs in the experimental group. The SSRI drugs were same as the control group.

##### 2.3.3.2 Control group

Patients used were treated with SSRIs in the control group.

#### 2.3.4 Types of outcome measurements

##### 2.3.4.1 Primary outcomes

The primary outcome are the Hamilton depressive disorder scale and clinical efficiency.

##### 2.3.4.2 Secondary outcomes

The primary secondary outcome are the self-rating depressive disorder scale, quality of life scale and Estradiol levels.

### 2.4 Collection and analysis of date

**Figure 1:**
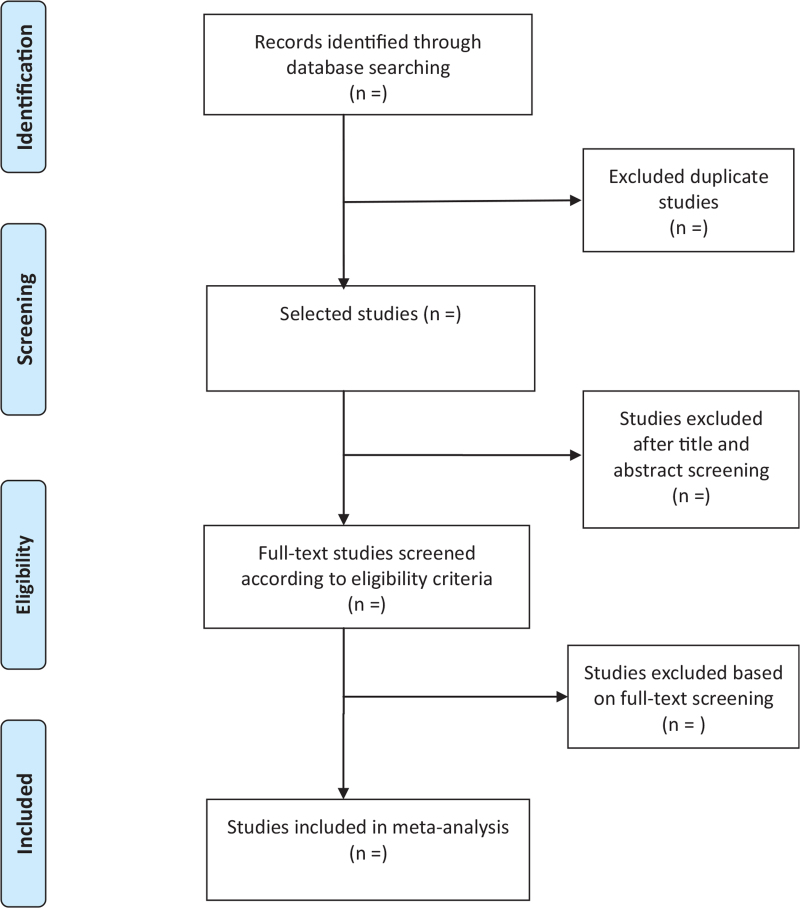
The preferred reporting items for systematic reviews and meta-analyses diagram.

We selected a PRISMA flow chart showing the processing of selected literature throughout the study (Fig. 1). Prior to screening the literature, all authors will discuss and determine the inclusion and exclusion criteria for the literature. After importing all the retrieved documents into EndNote software, V.X9 (United States), two authors independently reviewed and screened the literature based on the screening criteria. An initial screening was performed by reading the titles and abstracts of the literature for qualified studies and then excluding some duplicate studies or studies with incomplete information. Based on the results of the initial screening, the full text of the literature was downloaded and re-screened, and then studies that did not meet the screening criteria, such as those with missing important data and conflicting data, were excluded. After completing each step of the literature screening, 2 authors cross-check and if the 2 authors disagree, arbitration will be discussed with the third author.

#### 2.4.1 Data extraction

Two authors will independently extract the relevant information from included trials, such as first author, time of publication, group, sample size, patient age, intervention, duration of treatment, outcome indicators, method of randomization, blinding, concealment, allocation, and duration of follow-up. Gathering out specific information about acupuncture, such as the acupoints selected, the type of needles, the method of needle insertion, and the duration of needle retention according to the Standards for Reporting Interventions in Clinical Trials of Acupuncture (STRICA).^[18]^ A third author will help reconcile any differences between the 2 authors.

#### 2.4.2 Quality assessment

The Cochrane Collaboration tool was used to assess the risk of bias in randomized trials, including the generation of randomized sequences, allocation concealment, implementation of blinding (blinding of subjects and implementers and blinding of study outcomes), data integrity, selective publication, and other biases, all of which could be classified as “low risk”, “unclear” and ‘high risk”. “unclear” and “high risk”.^[18]^ After 2 authors have completed their assessments, the results are cross-checked if there is disagreement, a third author discusses arbitration.

#### 2.4.3 Dealing with missing data

If data from included studies are incomplete, we will try to contact the author of the article by sending an email or making a phone call. We will exclude studies with incomplete data if the author cannot be contacted.

#### 2.4.4 Data synthesis

We will use Review Manager Software (RevMan) V. 5.4 (Copenhagen, Denmark) for data analysis and quantitative data synthesis. If no statistical heterogeneity is found, a fixed-effects model is used for data synthesis. If there is significant statistical heterogeneity, we will use a random effects model.

#### 2.4.5 Assessment of heterogeneity

Heterogeneity in the forest diagram was determined by Q statistic or *I*^2^ statistic methods. When *P* < .1 or *I*^2^ > 50%, there was heterogeneity between studies; when *P* > .1 or *I*^2^ < 50%, there was no heterogeneity between studies.^[19]^

#### 2.4.6 Subgroup analysis

When heterogeneity occurs in the analysis, different drugs in SSRIs can be analyzed in subgroups.

#### 2.4.7 Assessment of reporting biases

In this analysis, once more than 10 trials were included, funnel plots could be used to test for reporting bias. When more than 20 studies were included, Egger regression was used to test reporting bias.

#### 2.4.8 Sensitivity analysis

Sensitivity analysis was performed to exclude tests with quality defects and to ensure the stability of analytical results based on sample size, study design, heterogeneity, methodological quality, and statistical models.

#### 2.4.9 Grading the quality of evidence

This study will use a quality-of-evidence rating method to assess the results obtained from this analysis. GRADE is generally applied to a large body of evidence. It has 4 evaluation levels, such as high, moderate, low, and very low. GRADE is used to assess the bias, inconsistency, discontinuity, and inaccuracy of test results. In the context of systematic reviews, quality reflects our confidence in the validity of the assessment.^[20]^

### 2.5 Ethics and dissemination

The approval of an ethical committee is not required to conduct this study. Patients and the public were not involved in the design and conception of this study. The results of this meta-analysis will be disseminated through publication in a peer-reviewed academic journal or relevant conference.

## 3 Discussion

The incidence of PDD is increasing with the increase of social pressure, but there are no clinically recommended drugs for the treatment of PDD. Medications and hormone replacement therapy are generally used to treat depressive disorder, but the results do not meet expectations and there are serious side effects, there is an urgent need to seek new treatment methods. The combination of acupuncture with SSRIs is not only synergistic, but also reduces the side effects associated with SSRIs.

The combination of acupuncture with SSRIs has been widely tried clinically and achieved good efficacy. The current lack of high-quality evidence validation has led to limited global replication. Although some potentially low-quality raw RCTs may affect the accuracy of the study, there is still a need to investigate them.

## Author contributions

**Conceptualization:** Zhan Gao.

**Data curation:** Tingting Song, Xiangju Gao.

**Formal analysis:** Zhan Gao, Mingzhou Gao.

**Funding acquisition:** Jieqiong Wang.

**Methodology:** Zhan Gao.

**Project administration:** Mingzhou Gao, Jie Gao, Mingqi Qiao.

**Supervision:** Jie Gao, Mingqi Qiao.

**Validation:** Zhan Gao, Jieqiong Wang, Mingzhou Gao, Mingqi Qiao.

**Writing – original draft:** Zhan Gao, Mingzhou Gao, Tingting Song.
